# Marital status and survival in patients with soft tissue sarcoma: A population‐based, propensity‐matched study

**DOI:** 10.1002/cam4.1802

**Published:** 2019-01-09

**Authors:** Shi‐long Zhang, Wen‐rong Wang, Ze‐juan Liu, Zhi‐ming Wang

**Affiliations:** ^1^ Department of Hematology, Zhongshan Hospital Fudan University Shanghai China; ^2^ Faculty of Physical Education Shandong Normal University Jinan China; ^3^ Department of Pharmacy, Maternal and Child Health Care Hospital of Zaozhuang Zaozhuang China; ^4^ Department of Medical Oncology, Zhongshan Hospital Fudan University Shanghai China

**Keywords:** marital status, soft tissue sarcoma, surveillance, epidemiology, and end results, survival analysis

## Abstract

**Background:**

Marital status serves as an independent prognostic factor for survival in a variety of cancers. However, its prognostic impact on soft tissue sarcoma (STS) has not yet been established.

**Objective:**

To investigate the impact of marital status on survival outcomes among STS patients.

**Methods:**

A total of 18 013 STS patients diagnosed between 2004 and 2015 were extracted from Surveillance, Epidemiology, and End Results (SEER) database. The marital status was classified into married, divorced, widowed, and single. Kaplan‐Meier analysis and multivariate Cox proportional hazards regression analysis were conducted to establish the impact of marital status on the overall survival (OS) and cancer‐specific survival (CSS). Subgroup analyses were conducted based on age, SEER historic stage and surgery condition. Propensity score matching (PSM) was used to perform a 1:1 matched‐pair analysis to minimize the group differences caused by covariates.

**Results:**

Married patients enjoyed better 5‐year overall survival (OS) and 5‐year cancer‐specific survival (CSS), compared with patients who were divorced, widowed, and single, respectively. Multivariate Cox proportional hazards regression analysis revealed that marital status was an independent prognostic and protective factor for survival among STS patients, and unmarried status was associated with higher mortality hazards for both OS and CSS. Additionally, widowed individuals had the highest risks of overall and cancer‐specific mortality compared to other unmarried groups. In the subgroup analyses, similar associations were also found. Furthermore, marital status still remained an independent prognostic and protective factor for both OS and CSS even in 1:1 matched‐pair analysis.

**Conclusions:**

Marital status was an independent prognostic and protective factor for survival for STS patients. Widowed patients suffered the highest death risks among the unmarried groups.

## 1 INTRODUCTION

Soft tissue sarcoma (STS) is a group of rare tumors.^1^ More than 50 histological subtypes have been identified, which vary in clinical manifestation, therapy, and prognosis.^2^ It accounts for approximately 1% of all solid tumors, with 5‐6 new cases per 100 000 people annually. And the 5‐year overall survival (OS) rate ranks from 55% to 65%.^3^, ^4^ Several factors are associated with poor prognosis, including age, pathological grade, tumor stage at diagnosis, and whether surgery or radiotherapy was performed or not. As the biopsychosocial medical model has been present, psychological and social factors have been more and more emphasized in cancers.^5^ Nowadays, multidisciplinary treatment has been the treatment principle for STS patients.^6^, ^7^, ^8^, ^9^ However, most clinicians mainly focused on pathophysiologic and clinical features, without addressing the impact of psychological and social factors on cancer.

Previous studies have shown that marital status is an independent prognostic factor for survival, and married patients tended to have better survival outcomes in esophageal cancer,^10^ primary liver cancer,^11^ gastric cancer,^12^ gallbladder cancer,^13^ pancreatic cancer,^14^ prostate cancer,^15^ and so on. However, some studies have also reported that the long‐term survival outcomes of cancer patients and marital status are not significantly correlated.^16^, ^17^, ^18^ Despite multiple studies on marital status and cancer prognosis, to our knowledge, neither retrospective nor prospective investigation has been performed to explore the association between marital status and prognosis among STS patients so far.

The aim of this study was to explore the impact of marital status on the survival of STS patients by virtue of the Surveillance, Epidemiology, and End Results (SEER) database, a US population‐based sample covering approximately 30% of the population in the United States.

## 2 MATERIALS AND METHODS

### 2.1 Data sources

Research data were extracted from the SEER database‐18 cohort database (Incidence ‐SEER 18 Regs Research Data + Hurricane Katrina Impacted Louisiana Cases, Nov 2017 Sub [1973‐2015 varying]), affiliating to the National Cancer Institute consists of 18 population‐based cancer registries, which represents nearly 26% of the US population.^19^ Published data about incidence, mortality, prevalence, survival, and marital status can be applied to assess the impact of cancer in the general population.

### 2.2 Inclusion and exclusion criteria

We extracted the information of patients diagnosed with STS between 2004 and 2015 from SEER database via SEER‐stat software (SEER*Stat 8.2.1). Patients were included when they met inclusion criteria as follow: (a) patients were aged 18 years or older at diagnosis; (b) the year of diagnosis was limited from 2004 to 2015; and (c) histological types were confined to fibromatous neoplasms (code: 8810, 8811, 8813, 8814, 8815, 8821, 8822, 8830, 8832, 8833, 8835, and 8836), rhabdomyosarcoma (8901, 8902, 8910, 8912, 8920, 8921, and 8991), specified (excluding Kaposi sarcoma; 8804, 8825, 8840, 8841, 8842, 8850, 8851, 8852, 8853, 8854, 8855, 8857, 8858, 8860, 8890, 8891, 8893, 8894, 8895, 8896, 8897, 8983, 8990, 8991, 9040, 9041, 9042, 9043, 9044, 9120, 9124, 9125, 9130, 9133, 9150, 9170, 9251, 9252, 9540, 9560, 9561, 9571, 9580, and 9581), Kaposi sarcoma (9140), and unspecified soft tissue sarcoma (8800, 8801, 8802, 8803, 8805, and 8806). The exclusion criteria included the patients: age <18 years, unknown marital status, domestic partner, incomplete follow‐up information, not the first tumor, all autopsy or death certificate cases, unknown survival time, and unknown cause of death.

### 2.3 Study variables

The following variables were extracted from the SEER database, including sex, age at diagnosis, race, year of diagnosis, marital status, pathological grade, tumor size, SEER historic stage, insurance record, surgery condition, vital status, cause of death, and months of survival. Marital status was described as four groups: married, divorced, single (never married), and widowed. STS patients are divided into two groups based on age at diagnosis (≤60 years vs >60 years). Patients diagnosed in different eras were also classified into three groups (2004‐2007, 2008‐2011, and 2012‐2015) to eliminate the survival differences resulting from therapeutic developments for STS over the past decades. Race was categorized as white, black, or others (American Indian/AK Native, Asian/Pacific Islander, and unknown race).

### 2.4 Statistical analyses

The baseline characteristics of the STS patients were presented with descriptive statistics. The categorical variables were expressed as frequency (percentages). The endpoints of this study were OS and CSS. Kaplan‐Meier survival analysis and a log‐rank test were used to compare survival difference between groups. Multivariable Cox proportional hazards regression analysis was used to identify the prognostic factors for survival outcomes among STS patients.

To minimize the covariates differences across groups, we conducted a 1:1 propensity score matching (PSM) analysis as follows: (a) Propensity scores were calculated for marital status (married and unmarried) for each patient with a binary logistic regression model that included all the aforementioned covariates; (b) using a nearest‐neighbor algorithm, we conducted 1:1 matching for married and unmarried patients with no replacement; (c) after matching, all baseline covariates between the two group before and after propensity scores were matched based on standardized difference(SD) of <0.1, which indicated that these covariates between the two groups were well‐balanced.

Kaplan‐Meier analysis, log‐rank test, and Cox proportional hazards regression were analyzed by R version 3.4.1 (https://www.r-project.org/). The R packages used in this study included *rms*,* survival*,* survminer*, and *ggplot2*. Propensity score matching was conducted by SPSS for Windows, version 22 (SPSS Inc, Chicago, IL, USA). All *P* values were two‐sided, and *P* < 0.05 was considered to be statistical significance.

## 3 RESULTS

### 3.1 Baseline demographic and clinical characteristics of STS patients

A total of 18 013 eligible STS patients diagnosed between 2004 and 2015 were identified in our study, including 9064 (50.32%) male and 8949 (49.68%) female patients. Details of demographic and clinical characteristics are summarized in Table 1. Among these patients, 10 791 (59.91%) were married, and 7222 (40.09%) were unmarried, including 1573 (8.73%) divorced, 1791 (9.94%) widowed, and 3858 (21.42%) single. In general, compared with married patients, the unmarried tended to be female, be diagnosed at later stage, and were less likely to undergo surgery. Among the unmarried patients, the single group had the highest proportions of men (10.95%), the most younger patients (16.79%), smaller tumor size (10.18%), localized stage (12.31%), higher pathological grade (7.87%), insured status (10.41%), and were more likely to receive surgery (18.74%). In addition, no differences were observed in the patients’ race, which had a SD of 0.058, and diagnosis year (SD: 0.024).

**Table 1:** Baseline demographic and clinical characteristics of STS patients according to marital status in SEER database

Characteristic	Total (%)	Married (%)	Unmarried (%)	Divorced (%)	Widowed (%)	Single (%)
	18 013 (100)	10 791 (59.91)	7222 (40.09)	1573 (8.73%)	1791 (9.94)	3858 (21.42)
Sex						
Male	9064 (50.32)	6015 (33.39)	3049 (16.93)	641 (3.56)	435 (2.41)	1973 (10.95)
Female	8949 (49.68)	4776 (26.52)	4173 (23.16)	932 (5.17)	1365 (7.53)	1885 (10.47)
Age						
≤60	10 011 (55.58)	5998 (33.30)	4013 (22.28)	837 (4.64)	152 (0.84)	3024 (16.79)
˃60	8002 (44.42)	4793 (26.61)	3209 (17.81)	736 (4.09)	1639 (9.10)	2297 (4.63)
Race						
White	14 338 (79.60)	8926 (49.55)	5412 (30.05)	1263 (7.01)	1453 (8.07)	2696 (14.97)
Black	2098 (11.65)	833 (4.63)	1265 (7.02)	210 (1.17)	195 (1.08)	860 (4.77)
Others^a^	1577 (8.75)	1032 (5.73)	545 (3.02)	100 (0.55)	143 (0.79)	302 (1.68)
Diagnosis year						
2004‐2007	5805 (32.23)	3499 (19.42)	2306 (12.80)	492 (2.73)	681 (3.78)	1133 (6.29)
2008‐2011	5793 (32.16)	3533 (19.62)	2260 (12.55)	494 (2.74)	553 (3.07)	1213 (6.74)
2012‐2015	6415 (35.61)	3759 (20.87)	2656 (14.74)	587 (3.26)	557 (3.09)	1512 (8.39)
Pathological grade						
Grade I	3513 (19.50)	2255 (12.52)	1258 (6.98)	278 (1.54)	259 (1.43)	721 (4.00)
Grade II	3166 (17.58)	1891 (10.50)	1275 (7.08)	254 (1.41)	268 (1.49)	753 (4.18)
Grade III	4456 (24.74)	2578 (14.31)	1878 (10.43)	389 (2.16)	522 (2.90)	967 (5.37)
Grade IV	6878 (38.18)	4067 (22.58)	2811 (15.60)	652 (3.62)	742 (4.12)	1417 (7.87)
Tumor size						
≤10 cm	8906 (49.44)	5470 (30.37)	3436 (19.08)	732 (4.06)	871 (4.83)	1833 (10.18)
10‐20 cm	5148 (28.58)	3013 (16.72)	2135 (11.85)	490 (2.72)	504 (2.80)	1141 (6.34)
>20 cm	2280 (12.66)	1334 (7.41)	946 (5.25)	208 (1.16)	224 (1.24)	514 (2.85)
Unknown	1679 (9.32)	974 (5.41)	705 (3.91)	143 (0.79)	192 (1.07)	370 (2.05)
SEER historic stage						
Localized	10 723 (59.53)	6570 (36.48)	4153 (23.06)	898 (4.99)	1037 (5.76)	2218 (12.31)
Regional	4532 (25.16)	2698 (14.97)	1834 (10.18)	406 (2.25)	476 (2.64)	952 (5.29)
Distant	2758 (15.31)	1523 (8.46)	1235 (6.85)	269 (1.49)	278 (1.54)	688 (3.82)
Insurance status						
Insured	11 068 (61.45)	7236 (40.17)	3832 (21.27)	910 (5.05)	1046 (5.81)	1876 (10.41)
Any Medicaid	1881 (10.44)	655 (3.64)	1226 (6.80)	221 (1.23)	185 (1.04)	820 (4.55)
Uninsured	604 (3.35)	222 (1.23)	382 (2.12)	66 (0.37)	26 (0.14)	290 (1.62)
Unknown	4460 (24.76)	2678 (14.87)	1782 (9.90)	376 (2.09)	534 (2.96)	872 (4.84)
Surgery						
No	2120 (11.77)	1105 (6.14)	1015 (5.63)	219 (1.22)	313 (1.74)	483 (2.68)
Yes	15 893 (88.23)	9686 (53.77)	6207 (34.46)	1573 (7.52)	1478 (8.20)	3375 (18.74)

a Included American Indian/Alaska Native, Asian/Pacific Islander, and unknown race.

### 3.2 Impact of marital status on overall survival (OS) and cancer‐specific survival (CSS) among STS patients.

**Figure 1:**
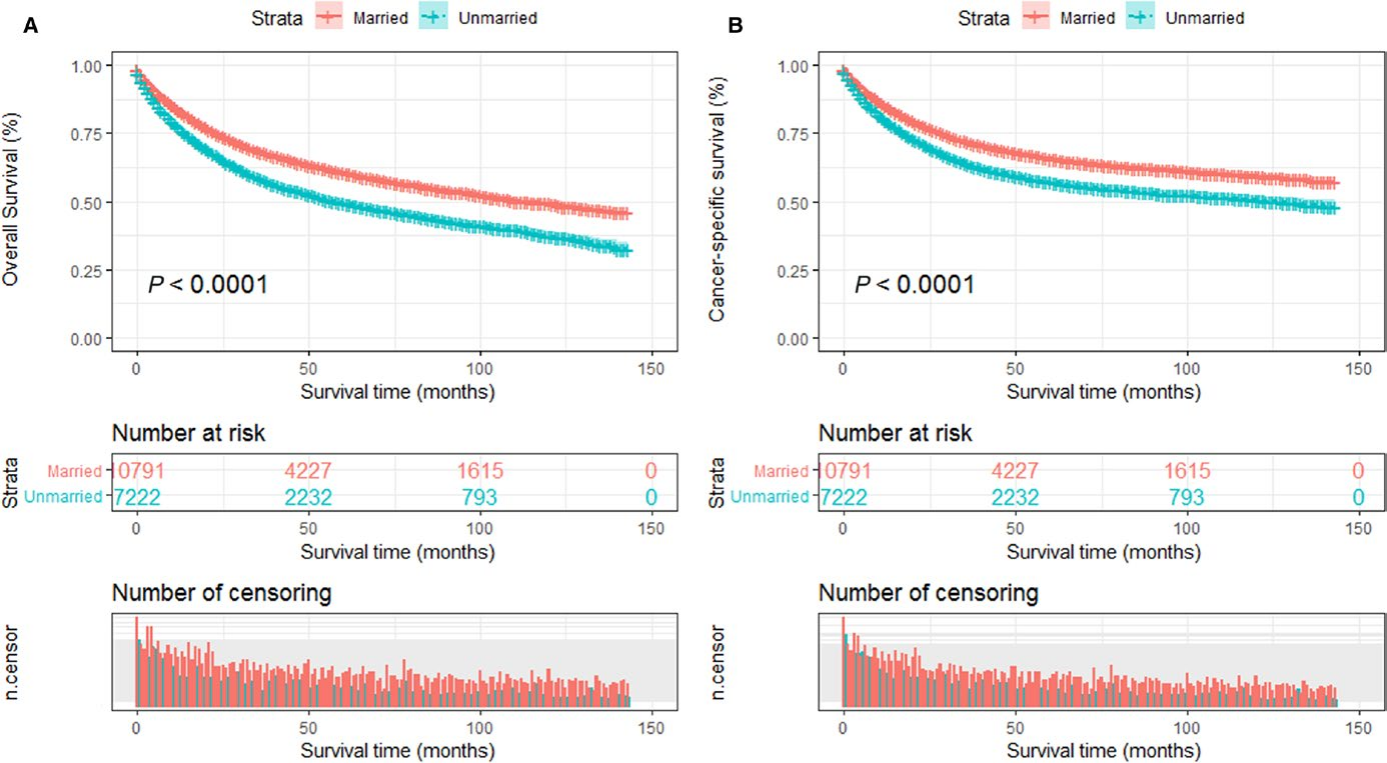
Kaplan‐Meier survival plots of STS patients according to marital status. A, Overall survival; B, cancer‐specific survival

We performed a Kaplan‐Meier analysis and log‐rank test to reveal the difference in overall survival (OS) according to marital status. The married patients had a better 5‐year OS rate than those unmarried (63.4% vs 49.5%, *P* < 0.001). These survival differences were also significant in the log‐rank test (Figure 1A). After adjustment for potential confounders such as sex, age, race, diagnosis year, pathological grade, tumor size, SEER historic stage, and surgery condition, multivariate Cox proportional hazards regression analysis demonstrated that marital status was an independent and protective factor for OS. And compared with married patients (as the reference group), unmarried patients had higher risks of mortality for OS (HR: 1.28, 95% CI: 1.22‐1.34, *P* < 0.001; Table 2).

**Table 2:** Univariate and multivariate survival analysis for evaluating the impact of marital status on overall survival (OS) among STS patients

Variables	5‐y OS	Univariate analysis		Multivariate analysis		
		Log‐Rank *χ* ^2^	*P*	HR	95% CI	*P* value
Marital status						
Married	63.4%	217	<0.001	Reference		
Unmarried	49.5%			1.28	1.22‐1.34	<0.001
Sex						
Male	57.4%	5.3	0.02	Reference		
Female	50.5%			0.96	0.92‐0.99	0.02
Age						
≤60	63.5%	636	<0.001	Reference		
˃60	47.1%			1.89	1.79‐1.98	<0.001
Race						
White	60.0%	29.7	<0.001	Reference		
Black	50.4%			1.06	0.98‐1.14	0.12
Others	56.3%			0.93	0.85‐1.01	0.09
Diagnosis year						
2004‐2007	56.2%	21	<0.001	Reference		
2008‐2011	58.7%			0.95	0.87‐1.03	0.21
2012‐2015	60.5%			0.89	0.81‐0.97	0.01
Pathological grade						
Grade I	84.3%	2085	<0.001	Reference		
Grade II	74.1%			1.71	1.52‐1.89	<0.001
Grade III	46.1%			3.71	3.38‐4.07	<0.001
Grade IV	42.2%			3.75	3.43‐4.10	<0.001
Tumor size						
≤10 cm	65.2%	613	<0.001	Reference		
10‐20 cm	50.3%			1.47	1.39‐1.55	<0.001
>20 cm	46.6%			1.67	1.55‐1.79	<0.001
SEER historic stage						
Localized	70.8%	5706	<0.001	Reference		
Regional	49.3%			1.63	1.54‐1.73	<0.001
Distant	12.3%			3.80	3.57‐4.05	<0.001
Insurance status						
Insured	58.1%	142	<0.001	Reference		
Any Medicaid	43.1%			1.31	1.21‐1.42	<0.001
Uninsured	54.8%			1.14	0.99‐1.31	0.06
Surgery						
Yes	11.7%	4883	<0.001	Reference		
No	62.0%			0.33	0.31‐0.37	<0.001

Similar to OS, the Kaplan‐Meier survival curve showed that cancer‐specific survival (CSS) among patients with different marital statuses was significant (log‐rank test *P* < 0.001; Figure 1B). The 5‐year CSS rate of married patients was 65.8%, which was higher than that of unmarried patients (57.1%; *P* < 0.001). In the multivariate Cox proportional hazards regression analysis, a similar trend was observed that marital status served as an independent prognostic and protective factor for CSS and unmarried patients suffered higher death risks of CSS than those who are married (HR: 1.19, 95% CI: 1.13‐1.26, *P* < 0.001; Table 3).

**Table 3:** Univariate and multivariate survival analysis for evaluating the impact of marital status on cancer‐specific survival (CSS) among STS patients

Variables	5‐y CCS	Univariate analysis		Multivariate analysis		
		Log‐Rank *χ* ^2^	*P*	HR	95% CI	*P* value
Marital status						
Married	65.8%	131	<0.001	Reference		
Unmarried	57.1%			1.19	1.13‐1.26	<0.001
Sex						
Male	65.5%	24.4	<0.001	Reference		
Female	60.4%			1.03	0.98‐1.09	0.190
Age						
≤60	65.8%	174	<0.001	Reference		
˃60	57.1%			1.49	1.42‐ 1.57	<0.001
Race						
White	54.3%	40.1	<0.001	Reference		
Black	60.0%			1.05	0.97‐1.14	0.168
Others	58.5%			0.97	0.89‐1.07	0.551
Diagnosis year						
2004‐2007	62.5%	1.8	0.4	Reference		
2008‐2011	61.9%			0.95	0.86‐1.05	0.319
2012‐2015	65.0%			0.89	0.81‐0.99	0.042
Pathological grade						
Grade I	91.2%	2182	<0.001	Reference		
Grade II	80.3%			2.34	2.03‐2.69	<0.001
Grade III	48.8%			5.74	5.06‐6.49	<0.001
Grade IV	47.9%			5.89	5.22‐6.66	<0.001
Tumor size						
≤10 cm	71.9%	693	<0.001	Reference		
10‐20 cm	55.8%			1.58	1.49‐1.68	<0.001
>20 cm	52.0%			1.84	1.69‐1.99	<0.001
SEER historic stage						
Localized	77.8%	6332	<0.001	Reference		
Regional	51.9%			1.88	1.76‐2.01	<0.001
Distant	13.9%			4.47	4.16‐4.79	<0.001
Insurance status						
Insured	64.3%	124	<0.001	Reference		
Any Medicaid	50.1%			1.26	1.15‐1.37	<0.001
Uninsured	59.3%			1.08	0.93‐1.26	0.281
Surgery						
Yes	15.3%	4763	<0.001	Reference		
No	68.2%			0.32	0.31‐0.35	<0.001

In addition, age, pathological grade, tumor size, SEER historic stage, and surgery condition were also confirmed as independent prognostic factors for both OS and CSS in the multivariate Cox proportional hazards regression analysis. The detailed description of each prognostic factor is listed in Tables 2 and 3.

### 3.3 Impact of different unmarried statuses on overall survival (OS) and cancer‐specific survival (CSS) among the STS patients

**Figure 2:**
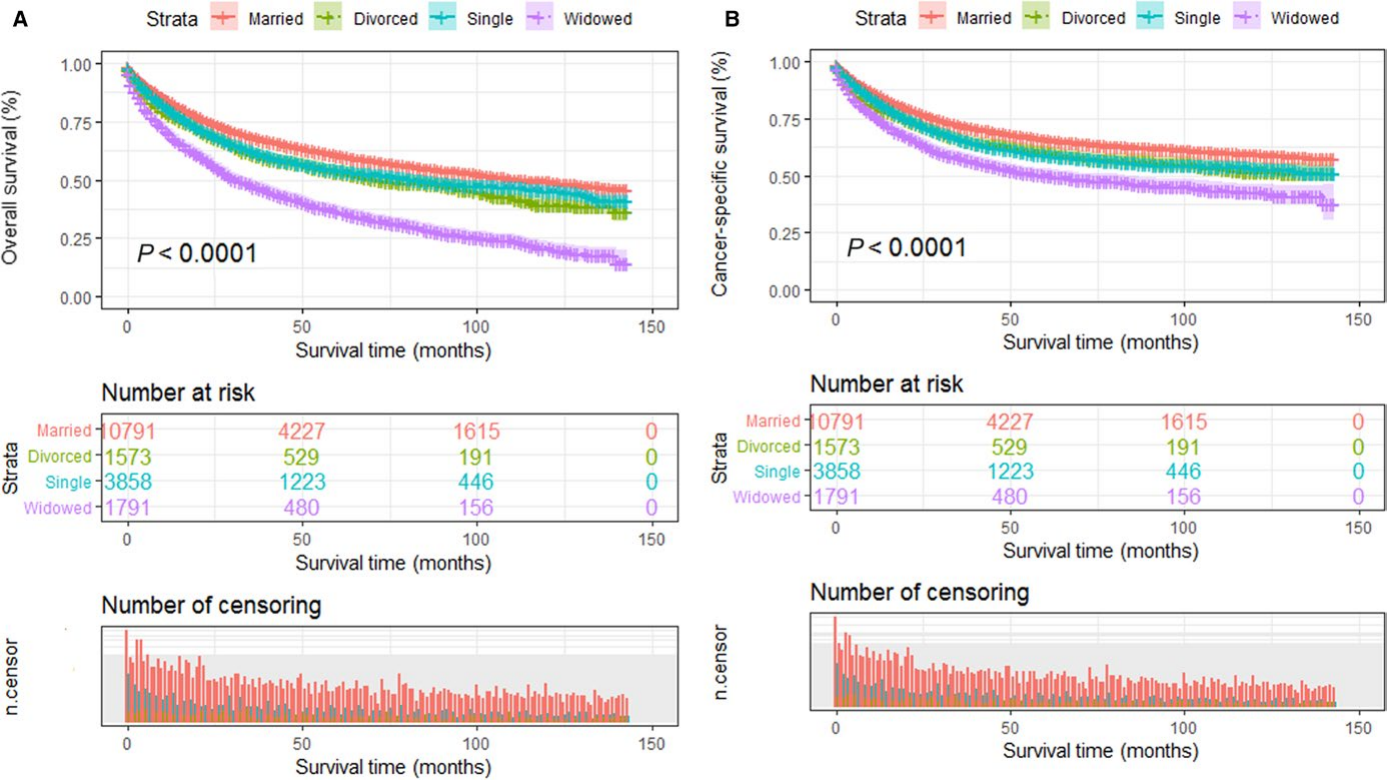
Kaplan‐Meier survival plots of the STS patients among married, divorced single, and widowed patients. A, Overall survival; B, cancer‐specific survival

Furthermore, in order to investigate whether different unmarried statuses could contribute to worse prognosis than being married, we divided unmarried patients into three subgroups: divorced, widowed, and single. Kaplan‐Meier survival analysis showed the 5‐year OS rate was 60.7% in the married group, 53.4% in the divorced group, 36.7% in the widowed group, and 54.3% in the single group. Widowed patients had the lowest rate of survival and the shortest survival time (*P* < 0.001; Figure 2A). After adjustment for other factors via the multivariate Cox proportional hazards regression analysis, divorced patients (HR: 1.18; 95% CI: 1.09‐1.28; *P* < 0.001), widowed patients (HR: 1.58; 95% CI: 1.47‐1.69; *P* < 0.001), and single patients (HR: 1.26; 95% CI: 1.18‐1.33; *P* < 0.001) all had worse OS than married patients (Table 4).

**Table 4:** Univariate and multivariate survival analysis for evaluating the impact of different unmarried statues on overall survival (OS) among STS patients

Variables	5‐y OS	Univariate analysis		Multivariate analysis		
		Log‐Rank *χ* ^2^	*P*	HR	95% CI	*P* value
Marital status						
Married	60.7%	131	<0.001	Reference		
Divorced	53.4%			1.18	1.09‐1.28	<0.001
Widowed	36.7%			1.58	1.47‐1.69	<0.001
Single	54.3%			1.26	1.18‐1.33	<0.001

Regarding CSS, the 5‐year CSS rate of the married patients was 65.8%, while those of the divorced, widowed, and single patients was 60.0%, 50.1%, and 59.1%, respectively. And among those unmarried patients, widowed patients had the worst OS outcomes (Figure 2B). After controlling other baseline characteristics, divorced patients (HR: 1.15; 95% CI: 1.05‐1.26; *P* < 0.001), widowed patients (HR: 1.19; 95% CI: 1.12‐1.26; *P* < 0.001), and single patients (HR: 1.38; 95% CI: 1.27‐1.51; *P* < 0.001) all had worse CSS than married patients (Table 5).

**Table 5:** Univariate and multivariate survival analysis for evaluating the impact of different unmarried statues on cancer‐specific survival (CSS) among STS patients

Variables	5‐y CSS	Univariate analysis		Multivariate analysis		
		Log‐Rank *χ* ^2^	*P*	HR	95% CI	*P* value
Marital status						
Married	65.8%	194	<0.001	Reference		
Divorced	60.0%			1.15	1.05‐1.26	0.002
Widowed	50.1%			1.19	1.12‐1.26	<0.001
Single	59.1%			1.38	1.27‐1.51	<0.001

### 3.4 Subgroup analysis of the effects of different marital statuses on overall survival (OS) among STS patients

Studies have reported the prognosis of STS patients is associated with age, clinical stage, and type of treatment.^20^ Our study has also identified several variables including age, SEER historic stage, and surgery condition as risk factors for STS mortality, based on multivariate Cox proportional hazards regression model analysis. Therefore, subsequently, we further explored the impact of different marital statuses on the OS of STS patients in the following subgroups that stratified by those variables. The results indicated that the marital status remained as an independent prognostic factor for better OS in most of the subgroups.

**Figure 3:**
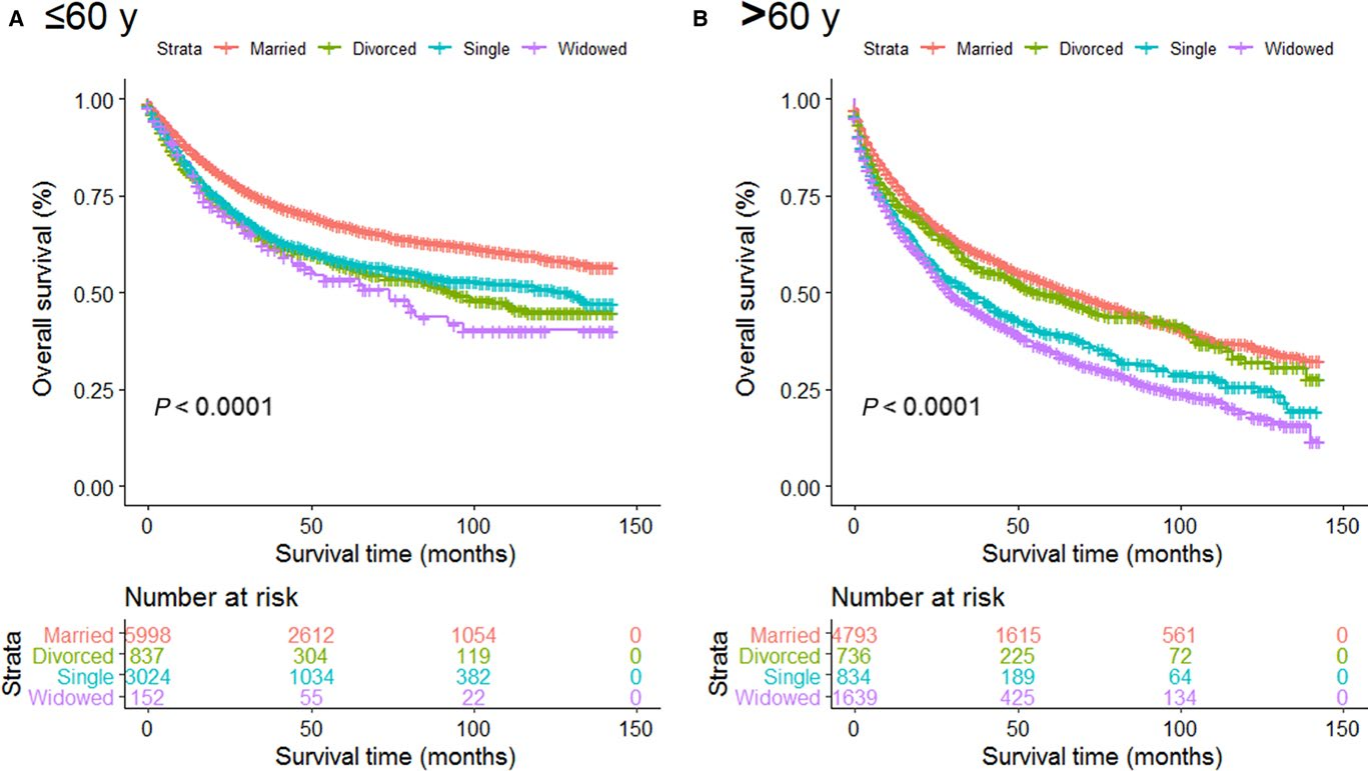
Survival curves for ST patients according to marital status in different age groups. A, Age ≤ 60 y; B, age > 60 y

**Figure 4:**
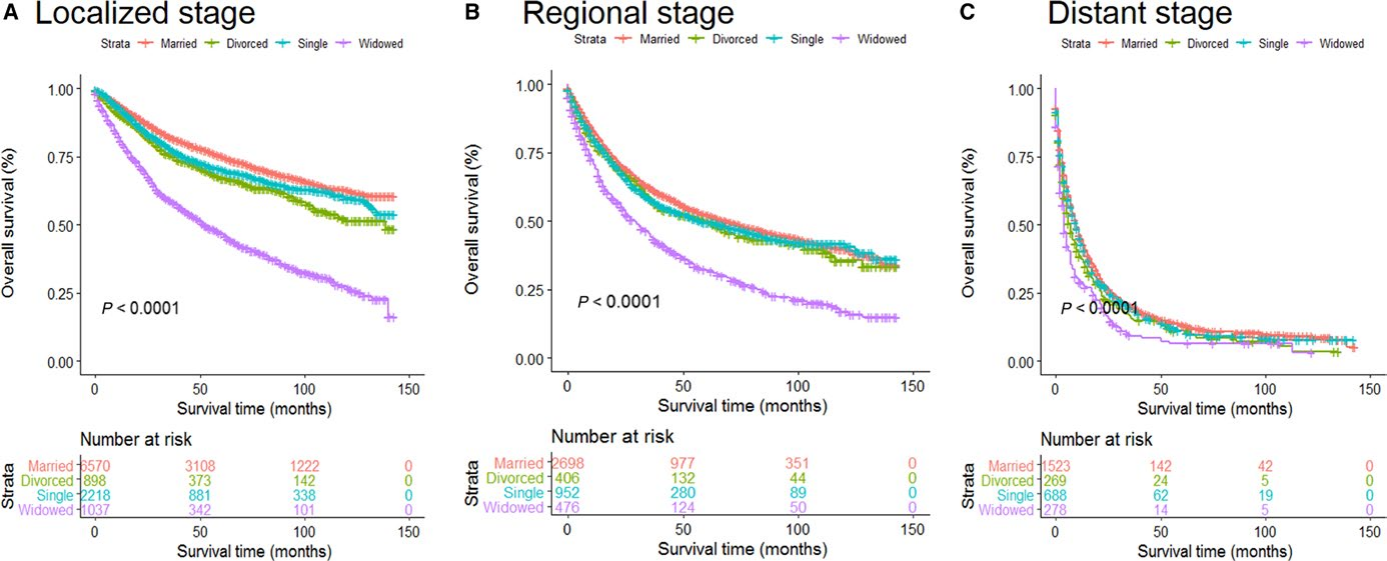
Survival curves for ST patients according to marital status in different disease stages. A, Localized stage; B, regional stage; and C, distant stage

**Figure 5:**
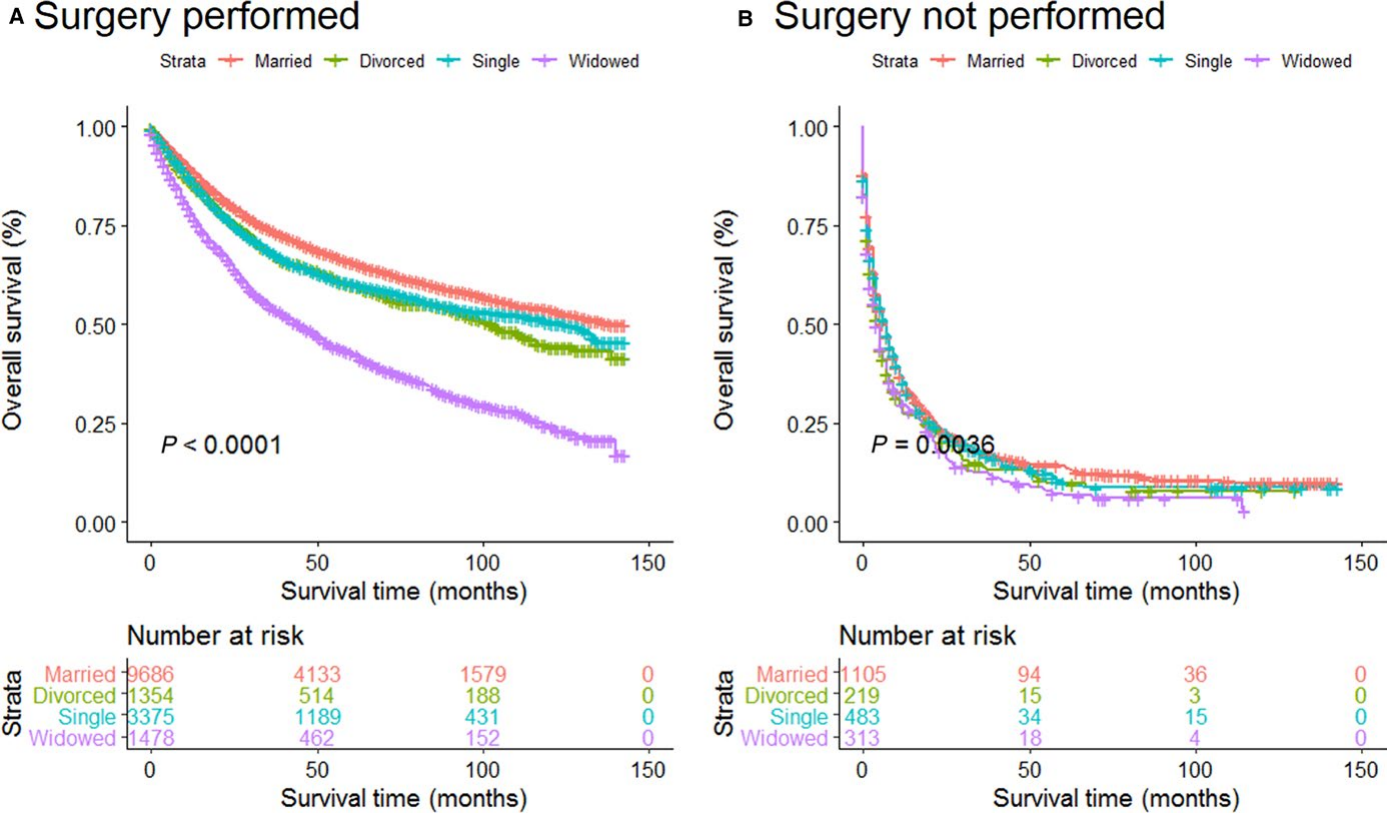
Survival curves for ST patients according to marital status in different surgery groups. A, Surgery performed; B, surgery not performed

Multivariate analysis showed that married patients of both age groups had better OS than other patients (all *P* < 0.001). The widowed suffered the highest risk mortality for OS, no matter they were older or younger than 60 years old (Figure 3A,B). Surprisingly, divorced older than 60 years old (*P *= 0.053) showed no survival differences from married patients of similar age range (Table 6). We then analyzed OS and HR based on different SEER historic stages, no matter which stage they were diagnosed, the married individuals enjoyed better survival outcomes than unmarried ones (Figure 4A‐C). Interestingly, when compared with married patients, divorced patients with regional or distant disease displayed no higher risk death for OS (Table 7). Furthermore, we stratified these patients by surgery condition. Consistent with previous results, married patients still enjoyed significant survival advantages and the widowed remained the highest death risks of OS in all the comparisons (Figure 5A,B, Table 8).

**Table 6:** Univariate and multivariate analysis for evaluating the impact of marital status on overall survival (OS) based on different age groups

Variables	5‐y OS	Univariate analysis		Multivariate analysis		
		Log‐Rank *χ* ^2^	*P*	HR	95% CI	*P*
Age						
=<60						
Married	67.3%	94.4	<0.001	Reference		
Divorced	56.9%			1.46	1.31‐1.65	<0.001
Widowed	53.3%			1.64	1.29‐2.09	<0.001
Single	58.2%			1.35	1.26‐1.46	<0.001
>60						
Marital status						
Married	52.3%	177	<0.001	Reference		
Divorced	49.3%			1.12	0.99‐1.25	0.053
Widowed	35.2%			1.65	1.52‐1.78	<0.001
Single	39.5%			1.44	1.30‐1.59	<0.001

**Table 7:** Univariate and multivariate analysis for evaluating the impact of marital status on overall survival (OS) based on different SEER stages

Variables	5‐y OS	Univariate analysis		Multivariate analysis		
		Log‐Rank *χ* ^2^	*P*	HR	95% CI	*P* value
*SEER stage*						
Localized						
Marital status						
Married	75.3%	459	<0.001	Reference		
Divorced	67.2%			1.36	1.21‐1.56	<0.001
Widowed	47.1%			2.78	2.52‐3.07	<0.001
Single	70.4%			1.18	1.07‐1.30	<0.001
Regional						
Marital status						
Married	52.3%	93.5	<0.001	Reference		
Divorced	49.8%			1.11	0.95‐1.29	0.264
Widowed	31.9%			1.81	1.59‐2.04	<0.001
Single	49.5%			1.07	0.96‐1.21	0.181
*Distant*						
Marital status						
Married	13.5%	36.8	<0.001	Reference		
Divorced	11.1%			1.19	1.03‐1.37	0.017
Widowed	6.7%			1.52	1.3‐1.74	<0.001
Single	11.9%			0.94	0.95‐1.17	<0.308

**Table 8:** Univariate and multivariate analysis for evaluating the impact of marital status on overall survival (OS) based on surgery condition

Variables	5‐y OS	Univariate analysis		Multivariate analysis		
		Log‐Rank *χ* ^2^	*P*	HR	95% CI	*P* value
*Surgery*						
Performed						
Marital status						
Married	65.9%	384	<0.001	Reference		
Divorced	60.1%			1.23	1.12‐1.35	<0.001
Widowed	42.9%			2.11	1.96‐2.28	<0.001
Single	32.9%			1.18	1.11‐1.25	0.024
Not performed						
Marital status						
Married	14.2%	13.5	0.004	Reference		
Divorced	8.8%			1.18	1.01‐1.39	0.036
Widowed	6.7%			1.26	1.10‐1.45	0.007
Single	9.9%			1.04	0.92‐1.17	0.051

### 3.5 Propensity score matching and survival analysis in 1:1 matched‐pair cohort for STS patients

In order to minimize the possible bias in baseline characteristics between married group and unmarried group and verify the accuracy and reliability of our results, we conducted a propensity score matching (PSM) in 1:1 matched‐paired cohort. The standard difference (SD) between all the baseline characteristics decreased significantly after PSM. Considering a SD <0.1 as a cutoff for balance, the distribution of sex, age, race, diagnosis year, SEER historic stage, insurance status, and surgery condition reached good balance. Finally, we obtained 10 028 patients including 5014 married patients and another 5014 unmarried ones. Table 9 summarizes the covariate balance between the married group and the unmarried group before and after PSM.

**Table 9:** Patient characteristics before and after propensity score matching (PSM)

Characteristic	Before matching			After matching		
	Married (%)	Unmarried (%)	Standard Difference	Married (%)	Unmarried (%)	Standard Difference
	10 791 (59.91)	7222 (40.09)		5014 (50%)	5014 (50%)	
Sex						
Male	6015 (33.39)	3049 (16.93)	0.135	2265 (22.58)	2278 (22.72)	−0.003
Female	4776 (26.52)	4173 (23.16)		2749 (27.41)	2736 (27.28)	
Age						
≤60	5998 (33.30)	4013 (22.28)	0.012	2806 (27.98)	2828 (28.20)	−0.004
˃60	4793 (26.61)	3209 (17.81)		2208 (22.02)	2186 (21.80)	
Race						
White	8926 (49.55)	5412 (30.05)	0.058	4246 (42.34)	4246 (42.34)	0.000
Black	833 (4.63)	1265 (7.02)		467 (4.65)	468 (4.67)	
Others	1032 (5.73)	545 (3.02)		301 (3.00)	300 (2.99)	
Diagnosis year						
2004‐2007	3499 (19.42)	2306 (12.80)	0.024	1644 (16.39)	1612 (16.07)	0.006
2008‐2011	3533 (19.62)	2260 (12.55)		1564 (15.60)	1600 (15.96)	
2012‐2015	3759 (20.87)	2656 (14.74)		1806 (18.01)	1802 (17.97)	
Pathological grade						
Grade I	2255 (12.52)	1258 (6.98)	0.081	903 (9.02)	916 (9.13)	−0.012
Grade II	1891 (10.50)	1275 (7.08)		868 (8.65)	873 (8.72)	
Grade III	2578 (14.31)	1878 (10.43)		1218 (12.14)	1217 (12.13)	
Grade IV	4067 (22.58)	2811 (15.60)		2025 (20.19)	2008 (20.02)	
Tumor size						
≤10 cm	5470 (30.37)	3436 (19.08)	0.053	2646 (26.39)	2667 (26.60)	−0.014
10‐20 cm	3013 (16.72)	2135 (11.85)		1429 (14.25)	1439 (14.35)	
>20 cm	1334 (7.41)	946 (5.25)		576 (5.74)	563 (5.61)	
Unknown	974 (5.41)	705 (3.91)		363 (3.62)	345 (3.44)	
SEER historic stage						
Localized	6570 (36.48)	4153 (23.06)	0.064	3175 (31.66)	3177 (31.68)	0.001
Regional	2698 (14.97)	1834 (10.18)		1195 (11.92)	1191 (11.88)	
Distant	1523 (8.46)	1235 (6.85)		644 (6.42)	646 (6.44)	
Insurance status						
Insured	7236 (40.17)	3832 (21.27)	0.169	3172 (31.63)	3136 (31.27)	0.003
Any medicaid	655 (3.64)	1226 (6.80)		370 (3.69)	396 (3.95)	
Uninsured	222 (1.23)	382 (2.12)		95 (0.95)	137 (1.37)	
Unknown	2678 (14.87)	1782 (9.90)		1377 (13.73)	1345 (13.41)	
Surgery						
No	1105 (6.14)	1015 (5.63)	−0.038	417 (4.16)	421 (4.20)	−0.001
Yes	9686 (53.77)	6207 (34.46)		4597 (45.84)	4593 (45.80)	

**Figure 6:**
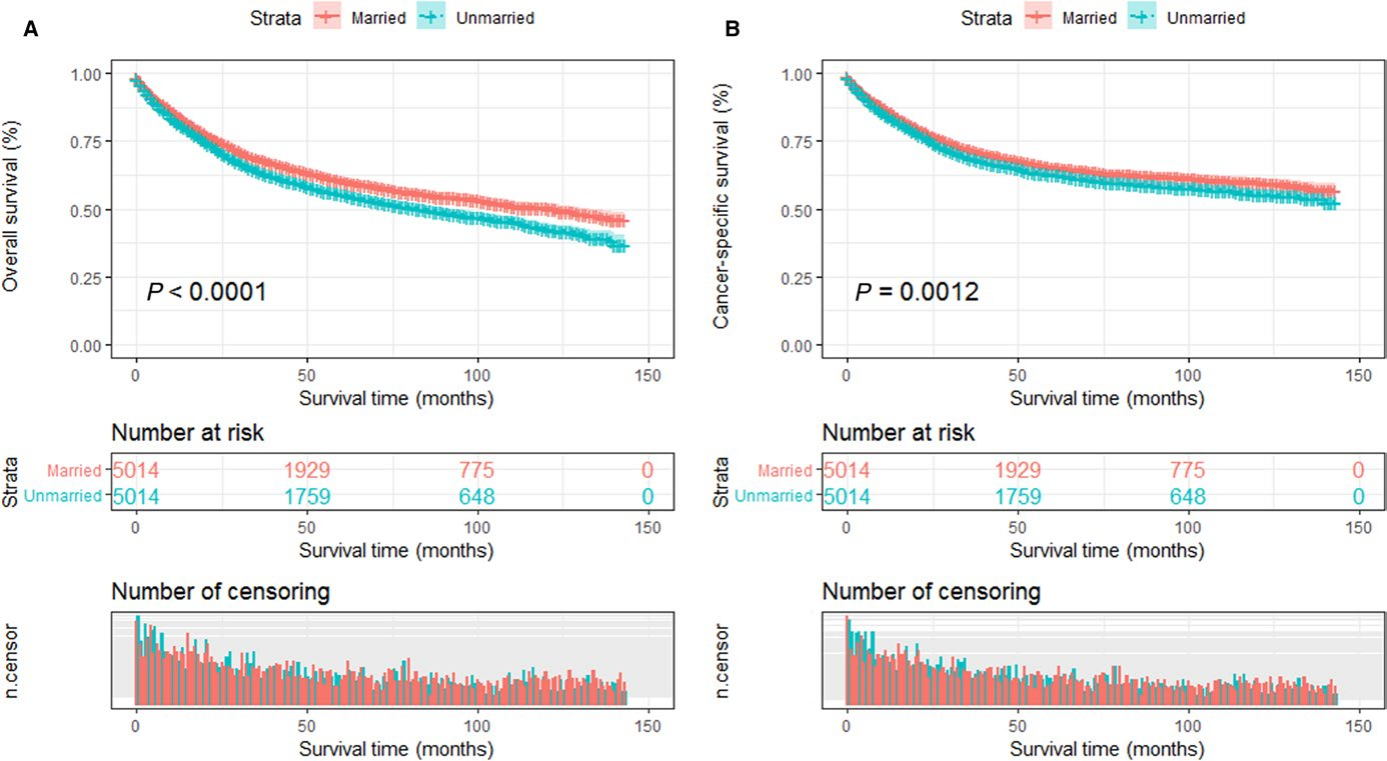
Kaplan‐Meier survival curves of the STS patients according to marital status after propensity score matching (PSM). A, Overall survival; B, cancer‐specific survival

Despite the similar basic characteristics, married patients still remained better survival outcomes than those unmarried in the Kaplan‐Meier survival analysis. The 5‐year OS rate was 60.3% in the married group and 55.4% in unmarried group (*P* < 0.001; Figure 6A). And likewise, the 5‐year CSS of married patients was 65.4%, which was significantly higher than those in unmarried group (62.7%; *P* < 0.001; Figure 6B).

To guarantee the reliability, we further performed univariate Cox proportional hazards regression analysis. Although the HR was not higher after PSM than before PSM, unmarried patients still suffered higher death risks of both OS (HR: 1.23; 95% CI: 1.16‐1.32; *P* < 0.0001) and CSS (HR: 1.17; 95% CI: 1.09‐1.25; *P* < 0.0001) than those who were married patients (Tables 10 and 11).

**Table 10:** Univariate analysis of overall survival (OS) for STS patients after propensity score matching (PSM)

Variables	5‐y OS		Univariate analysis	
		HR (95% CI)	Log‐rank *χ* ^2^	*P* value
Marital status				
Married	60.3%	Reference	30	<0.001
Unmarried	55.4%	1.23 (1.16‐1.32)		

**Table 11:** Univariate analysis of cancer‐specific survival (CSS) for STS patients after propensity score matching (PSM)

Variables	5‐y CSS		Univariate analysis	
		HR (95% CI)	Log‐Rank *χ* ^2^	*P* value
Marital status				
Married	65.4%	Reference	10.6	0.001
Unmarried	62.7%	1.17 (1.09‐1.25)		

## 4 DISCUSSION

Despite an uncommon cancer, our study was the largest one to investigate the impact of marital status on survival outcomes among STS population. For the first time, we found that marital status was an independent prognostic factor for STS patients and demonstrated that married patients showed significant survival advantages over unmarried patients, including divorced, widowed, and single patients. Particularly, subgroup analyses were performed based on several established risk factors in this study or others. And it showed marital status could independently predict mortality risk in most of the age, SEER historic stage, and surgery subgroups. Furthermore, in order to balance the covariates between the married and unmarried groups and verify our results, we performed a 1:1 propensity score matched analysis. After matching, marital status was confirmed again as an independent prognostic and protective factor for STS patients, and married patients enjoy lower overall and cancer‐specific risk mortality than the unmarried. In addition, age, pathological grade, tumor size, SEER historic stage, and surgery were also independent prognostic factors for STS patients, which were consistent with previous studies.^21^, ^22^, ^23^

Furthermore, our study indicated that younger STS patients (≤60 years) benefit more from marriage than older patients (>60 years) do. There are several potential reasons for this age discrepancy. First, marriage may grant more mental and financial support to younger than to older. Second, most youngers tend to have unhealthy habits such as smoking and drinking than elders do, indicating that youngers benefit more than elders from the lifestyle changes caused by marital status. In addition, the proportion of widowed elderly patients was the highest (9.1%) among those who were unmarried; this suggested that the poorest survival outcomes of the widowed individuals may also be associated with age.

Interestingly enough, several studies have reported that insurance status is associated with better prognosis in certain cancers.^11^, ^12^ In our study, we also demonstrated insurance status was an independent and protective factor for STS patients. And the proportion of patients with insurance was the highest in the single group, at 10.41% (compared with 5.05% in the divorced group and 5.81% in the widowed group). These results suggest to us that insurance is especially important for unmarried patients, which will provide more stability and security to them. However, compared with married people, unmarried patients, especially for single individuals, showed a distinct tendency to receive no surgery, partly resulting in their survival disadvantages.^24^ Early detection was closely associated with better prognosis for STS.^25^ However, in our study, this trend was less obvious since we conducted a 1:1 matched‐paired cohort analysis by PSM. After matching, the percentages of patients with localized and regional stage or distant metastasis were comparable between two groups. Therefore, survival benefits in the married group did not stem from the advantage of early detection.

Survival benefits associated with marriage have been supported by several studies; however, the underlying mechanisms have been not completely elucidated. A better explanation may lie in physiological psychology and socio‐economics. From a physiological psychology perspective, as a lethal disease which brings serious threat to human health, cancer damages physical health and leads to adverse psychological stress reaction at the same time. A cancer patient usually experiences higher levels of psychological stress and depression,^26^ and this can result in the imbalance of neuro‐endocrine‐immune network^27^ via glucocorticoid resistance and increased catecholamines.^28^ It has been well documented that glucocorticoid resistance and catecholamine could promote tumor growth, invasion, and metastasis through immunosuppressive actions.^29^, ^30^ In addition, extensive epidemiological evidence shows psychological stress also affects individual behaviors such as smoking, alcohol consumption, overeating, and drug abuse that in turn adversely affect an individual's health and mortality.^31^

For cancer patients, they often need more emotional supports than other patients. In addition to creating feelings of warmth and closeness, emotional support can help inoculate them from psychological stress and depression. Because of spouse emotional support and help, married patients display less stress and depression than their unmarried counterparts after a cancer was diagnosed.^32^ Kamen *et al*. conducted an analysis of 292 prostate cancer patients to explore the relationship between partner support and psychological stress. In their study, married patients with enough spouse support were more likely under lower levels of psychological distress than unmarried patients.^33^

The adherence to medical advice and therapy is also important for cancer patients. A recent meta‐analysis indicates that married patients usually have better adherence and tolerance with prescribed treatments and regular follow‐ups, partly through supports from their spouses.^34^ Increased psychological stress and less social support may result in worse cancer outcomes. Hence, under the current concept of multidisciplinary treatment for cancer, the clinical value of psychological intervention should be emphasized again. And perhaps more aggressive health education and reminder interventions should be taken regularly among unmarried patients.

From a socio‐economic perspective, a cancer diagnosis means not only physical and psychological suffering, but also any adverse impact on the economic burden. A large systematic review of researches published since 1995 was conducted to assess the association of cancer survival with socio‐economic status. And it indicated that married patients usually possess strong financial resources,^35^, ^36^ which made it easier to get access to expensive therapies and thus was associated with better prognosis. Besides, they may also get additional health care from their spouses. Otherwise, unmarried patients may tend to have economic difficulties, weaker ability to pay, but they must bear the same cost, so they should greater disease economic burden relatively.^37^ In our study, compared with unmarried individuals, the married had a larger percentage of surgery (53.77% vs 34.46%), which partly contributed to their survival advantages. It was at least plausible that disparities in survival outcomes among patients with different marital status were partly due to better access to the medical and health services.

In spite of the strengths of this study including large‐sized sample, subgroup analysis, and PSM method, there were some limitations that could not be ignored. First of all, SEER database does not provide details about the duration and happiness degree of the marriage, and marriage history, which weaken the ability to evaluate the impact of marriage on survival among STS patients. Most notably, socio‐economic factors that may affect either marital status or cancer survival are inaccessible in the SEER database on an individual level. In addition, the marital status of STS patients may change in the follow‐up period, which may interfere with the protective effect of marriage. Secondly, only legal marriage was recorded in the SEER database, and unrecorded marital status including gay, lesbian, bisexual, and transgender may biase our results. Thirdly, and perhaps the most important, several important clinical and treatment information closely associated with prognosis is not registered in SEER database, such as chemotherapy, types of surgery and radiotherapy, HIV infection status, and comorbidity.^20^ These missing factors may potentially confound our results. Nevertheless, despite these inevitable limitations, our study still demonstrated the importance of marital status, in significantly improving the survival in married STS patients.

Overall, our study had identified for the first time that marital status was an independent prognostic and protective factor for survival among STS patients and married patients enjoyed significant survival benefits than those unmarried. Especially, our study also showed that widowed patients are at the highest death risk compared with married patients. These results were further verified through PSM method. Physiological psychosocial and socio‐economic factors may contribute to the survival benefits associated with marriage. Therefore, timely psychological interventions and sufficient socio‐economic supports should be an essential component of multidisciplinary treatment for unmarried patients, especially widowed patients.

## ETHICAL APPROVAL

Data released from the SEER database do not require informed patient consent because it contains no personal identifying information and is publicly available for researchers worldwide. We got permission to access the research data in the SEER database by National Cancer Institute, USA, and the username was 13264‐Nov2017.

## CONFLICT OF INTEREST

All of the authors have no conflict of interests to declare.
